# Soil-transmitted helminth infection, anemia, and malnutrition among preschool-age children in Nangapanda subdistrict, Indonesia

**DOI:** 10.1371/journal.pntd.0009506

**Published:** 2021-06-17

**Authors:** Yenny Djuardi, Gilbert Lazarus, Difa Stefanie, Umi Fahmida, Iwan Ariawan, Taniawati Supali

**Affiliations:** 1 Department of Parasitology, Faculty of Medicine, Universitas Indonesia, Jakarta, Indonesia; 2 Faculty of Medicine, Universitas Indonesia, Jakarta, Indonesia; 3 Southeast Asian Ministers of Education Organization Regional Centre for Food and Nutrition (SEAMEO RECFON), Pusat Kajian Gizi Regional Universitas Indonesia, Jakarta, Indonesia; 4 Department of Biostatistics and Population Studies, Faculty of Public Health, Universitas Indonesia, Depok, Indonesia; Istituto Superiore di Sanità, ITALY

## Abstract

**Background:**

Soil-transmitted helminth (STH) infections are still prevalent in Indonesia, with roughly one-third of infected population being preschool-age children (PSC), which are generally at higher risk of morbidity such as malnutrition and anemia. This study aimed to investigate the association of STH infections with nutritional status and anemia among PSC in Nangapanda subdistrict, Ende, East Nusa Tenggara.

**Methods:**

A cross-sectional survey involving PSC ranging from 12 to 59 months old from Nangapanda subdistrict, Ende district, East Nusa Tenggara was performed. Socio-demographic, breastfeeding, and complementary feeding information was obtained from structured questionnaires, while nutritional and anemia status was determined from anthropometry and hemoglobin measurements, respectively. Anthropometric z-scores were calculated based on the World Health Organization 2006 standards and stool samples were examined using Kato-Katz method.

**Results:**

A total of 393 PSC randomly selected from 22 villages were examined. The prevalence of underweight, stunting, wasting, and anemia were 33.1%, 40.2%, 17.1%, and 60.3%, respectively. STH infection, predominated by *Ascaris lumbricoides*, was found in 160 (58.8%) PSC. Single STH infection, but not multiple infection, was independently associated with a lower risk of anemia (odds ratio [OR] 0.320, 95% confidence interval [CI]: 0.126–0.809, p = 0.016). Similar association with anemia was also found on mild STH infection (OR 0.318 [95% CI: 0.114–0.887], p = 0.029). On the other hand, younger children were found to have a higher risk of anemia and stunting. None of the examined variables were independently associated with underweight and wasting.

**Conclusion:**

STH infection as well as anemia and malnutrition were prevalent in this region. However in this study, current STH infections seemed to have minimal negative impact on children’s nutritional status.

## Author summary

Soil-transmitted helminth (STH) infection, which frequently coexist with anemia and malnutrition, remains a major disease burden to preschool-age children (PSC) as they may bring negative impacts on children’s growth and development. In this cross-sectional study, we investigated the prevalence of STH infection in Nangapanda subdistrict in Indonesia and explored the association between STH infection with poor nutritional status and anemia among PSC. Poor nutritional status was classified into underweight, stunting, and wasting, while anemia was defined as hemoglobin levels below 11 gr/dL. We revealed that many PSC in this area suffered from STH infection (primarily by *Ascaris lumbricoides*), malnutrition, and anemia. We also discovered that younger PSC were more vulnerable to stunting and anemia, whereas current single and mild STH infection was independently associated with a lower risk of anemia. Although no evidence for negative impact of STH infection was found in this study, the high prevalence of STH infection, malnutrition, and anemia in the study population warrants urgent intervention in order to improve the children’s health and wellbeing. Further research is required to assess the quality and quantity of food intake, as well as to investigate socioeconomic and environmental factors contributing to the poor nutritional status.

## Introduction

Soil-transmitted helminths (STH) infection, a neglected tropical disease caused by *Ascaris lumbricoides*, *Trichuris trichiura*, and hookworms, are commonly found worldwide, especially in tropical and subtropical regions [1]. It is associated with a disease burden of over 3.3 million disability-adjusted life years [2] and a global prevalence of 1.7 billion cases [1]. In terms of prevalence, Indonesia ranked second (70,642,364 cases), one-third of which occurred in preschool-age children (PSC) [3]. Among all provinces in Indonesia, South Sulawesi, Banten, DKI Jakarta, Bali, Papua, and Nusa Tenggara are known for their severe endemicity [4]. In East Nusa Tenggara itself, about 66% of adults were infected by at least 1 type of STH, comprising of mainly hookworms (51.7%) and followed by *A*. *lumbricoides* (21.8%) and *T*. *trichiura* (19.7%) [5].

While STH infection may occur in all age groups, PSC are at higher risks of mortality and morbidity [6]. Morbidities associated with STH infection in this age group include anemia and malnutrition; which, in the long run, may cause retarded growth and neurocognition leading to poor school performance and attendance as well as decreased productivity [7]. Besides STH infection, it is also important to note that several other etiological factors, including the quality and quantity of nutrient intake as well as environmental enteropathy, may also contribute to malnutrition and anemia [8]. Since East Nusa Tenggara is one of the provinces in Indonesia with a high prevalence of wasting and stunting [9], accompanied by the fact that STH infection and child malnutrition frequently coexist, this study aimed to investigate the association of STH infections with nutritional status as well as anemia in PSC in Nangapanda subdistrict, Ende, East Nusa Tenggara.

## Methods

### Ethics statement

Written informed consent for the children to participate in this study was obtained from their parents or guardians. This study protocol has been approved by Research Ethical Committee, Faculty of Medicine Universitas Indonesia-Cipto Mangunkusumo National General Hospital (653/UN2.F1/ETIK/2014).

### Study design, location, and participants

This study is part of a larger study (“Improving the health quality based on health education in Nangapanda subdistrict, East Nusa Tenggara in Nangapanda subdistrict, Ende, NTT”) conducted by the Department of Parasitology, Faculty of Medicine, Universitas Indonesia, in collaboration with the South East Asian Ministers of Education Regional Centre for Food and Nutrition (SEAMEO RECFON) and the Faculty of Public Health, Universitas Indonesia. A cross-sectional survey between December 2013 and January 2014 was performed by involving PSC with age ranging from 12 to 59 months residing in Nangapanda subdistrict, Ende, East Nusa Tenggara, Indonesia, which has been previously reported as an endemic area of STH infections [4]. Nangapanda is a rural area with an approximate population of 22,000 people scattered over 22 villages [10]. A minimum sample size of 385 was calculated based on estimation of proportion [11], with p = 50% for the prevalence of stunting in East Nusa Tenggara [12], absolute precision of 5% and α = 0.05. The participants were selected by a clustered random sampling method; each village was considered as a cluster and 18 children were randomly selected from each cluster.

### Data and sample collection

#### Demographical and anthropometric data

Demographical data consisting of PSC’s age, sex, and breastfeeding status, as well as maternal education level were obtained from their mothers using a questionnaire during house-to-house visits. Breastfeeding status was dichotomized into exclusive and non-exclusive breastfeeding. Exclusive breastfeeding was defined as the sole provision of breast milk for the first six months of life [13,14] and was further stratified according to the initiation time of complementary feeding. Timely complementary feeding was defined as the introduction of solid, semi-solid, or soft foods to PSC between 6–8 months old, while late complementary feeding was defined as the introduction of the aforementioned nourishments beyond eight months [14,15]. Non-exclusive breastfeeding was determined when breastfeeding practice was discontinued or complementary feeding was introduced before six months of age [13,14].

For anthropometric measurements, body weight was measured using a seca weighing scale, while body length using a seca 213 mobile stadiometer with 0.1 cm precision (seca Deutschland, Hamburg, Germany). Anthropometric parameters consisting of weight-for-age z-score (WAZ), height-for-age z-score (HAZ), and weight-for-height z-score (WHZ) were calculated using the World Health Organization (WHO)-Anthro ver. 3.2.2. [16]. WAZ, HAZ and WHZ score of < -3 SD, were classified as severely underweight, severely stunted, or severely wasted, respectively; score of ≥ -3 SD to < -2 SD were classified as underweight, stunted or wasted, respectively; and score of ≥ -2 SD was classified as normal [17].

#### Examination of blood and stool samples

A total of 150 μl finger blood sample was collected in BD Microtainer blood collection tube with ethylenediaminetetraacetic acid (EDTA) as anticoagulant (Becton, Dickinson and Company, New Jersey, USA). The blood samples were subsequently brought to the laboratory for assessment of hemoglobin level using HemoCue Hb 201 (HemoCue AB, Ängelholm, Sweden). Anemia was defined by hemoglobin level less than 11 g/dL in children and less than 12 g/dL in mothers [18].

Fresh stool samples were collected from PSC in labeled stool containers. The parents were given labeled stool containers and were instructed to provide fresh stool samples from PSC in the morning. The stool samples were collected by the research team every morning through home visits and were transferred to the field laboratory within three hours from the collection. The samples were then processed by Kato-Katz method and the slides were read by microscopic examination within one hour to avoid damage to the hookworm eggs. The number of eggs per species were subsequently converted into eggs per gram (epg) of feces by multiplying the number of eggs per slide with 24. The intensity of infection was classified according to the WHO criteria with cut-off values as following: *A*. *lumbricoides*, light (1–4999 epg), moderate (5000–49,999 epg), and heavy (≥50,000 epg); hookworm, light (1–1999 epg), moderate (2000–3999 epg) and heavy (≥4000 epg); *T*. *trichiura*, light (1–999 epg), moderate (1000–9999 epg) and heavy (≥10,000 epg) [19].

### Statistical analysis

Baseline data were expressed as means (standard deviations [SD]) or medians (interquartile ranges [IQR]), depending on the normality of data distribution as analyzed by Kolmogorov-Smirnov tests. Pearson’s chi-squared and Fischer’s exact tests were utilized to examine whether the prevalence and intensity of STH infection, poor nutritional status, and anemia significantly differed across age groups and sex. To assess the predictors of nutritional and anemia status, univariate and multivariate logistic regressions were performed. In the regressions, the nutritional status was classified into binary outcomes: normal and stunting for HAZ, normal and underweight for WAZ, and normal and wasting for WHZ. Variables associated with each outcome at p<0.20 were included in the multivariate analysis. Sex and age of PSC were also included as a priori covariates in the multivariate regression regardless of their significance in the univariate model [20]. All data were analyzed in SPSS 24.0 (SPSS Inc., Chicago, Ill) and a p-value of <0.05 indicated statistical significance.

## Results

### Characteristics of study population

A total of 404 PSC from 22 villages were eligible for enrollment in this study. Of these, 393 PSC had completed questionnaires (response rate: 97.28%). Demographic and health characteristics of the enrolled PSC and their mothers are shown in **Table 1**. Two-hundred and six (52.4%) participants were boys, and the median age was 31.6 months (IQR 22.4–44.2). Among them, 240 PSC (64.2%) were exclusively breastfed till 6 months of age and began to receive complementary feeding since 6–8 months of age, whereas only 10 children (2.7%) were never breastfed. Most of the mothers were primary and high school graduates (42.1% and 41.1%, respectively), and 111 (49.6%) of the mothers were anemic.

**Table 1:** Characteristics of enrolled preschool-age children (PSC).

Characteristics of participants	N (%)
Age (months)	N = 393
12–23	120 (30.5)
24–35	113 (28.8)
36–47	90 (22.9)
48–60	70 (17.8)
Sex	N = 393
Boys	206 (52.4)
Girls	187 (47.6)
Maternal education level	N = 392
None	41 (10.5)
Primary	165 (42.1)
Secondary	161 (41.1)
Higher education	25 (6.4)
Maternal hemoglobin status	N = 224
Anemia	111 (49.6)
Normal	113 (50.4)
Breastfeeding status	N = 374
Never breastfeed	10 (2.7)
Exclusive + Timely complementary feeding	240 (64.2)
Exclusive + Late complementary feeding	30 (8.0)
Non-exclusive	94 (25.1)
Number of helminth species	N = 272
None	112 (41.2)
*A*. *lumbricoides*	51 (18.8)
*T*. *trichiura*	28 (10.3)
Hookworm	3 (1.1)
Al+Tt	56 (20.6)
Al+Hw	6 (2.2)
Al+Tt+Hw	16 (5.9)
Any infection	160 (58.8)
Severity of helminth infection	
*A*. *lumbricoides*	129 (47.4)
Mild	34 (26.4)
Moderate	64 (49.6)
Severe	31 (24.0)
*T*. *trichiura*	100 (36.8)
Mild	68 (68.0)
Moderate	28 (28.0)
Severe	4 (4.0)
Hookworm	25 (9.2)
Mild	24 (96.0)
Moderate	0 (0.0)
Severe	1 (4.0)
Nutritional status	N = 393
Weight-for-age (WAZ)	
Severely underweight	34 (8.7)
Underweight	96 (24.4)
Normal	263 (66.9)
Height-for-age (HAZ)	
Severely stunted	55 (14.0)
Stunted	103 (26.2)
Normal	235 (59.8)
Weight-for-height (WHZ)^#^	
Severely wasted	11 (2.8)
Wasted	56 (14.3)
Normal	325 (82.9)
Hemoglobin status	N = 224
Anemia	135 (60.3)
Normal	89 (39.7)

^#^WHZ score was missing in one preschool-age children. PSC, preschool-age children; Al, *Ascaris lumbricoides;* Tt, *Trichuris trichiura*; Hw, hookworm; WAZ, weight-for-age z-score; HAZ, height-for-age z-score; WHZ, weight-for-height z-score.

### Prevalence and intensity of STH infection

**Fig 1:**
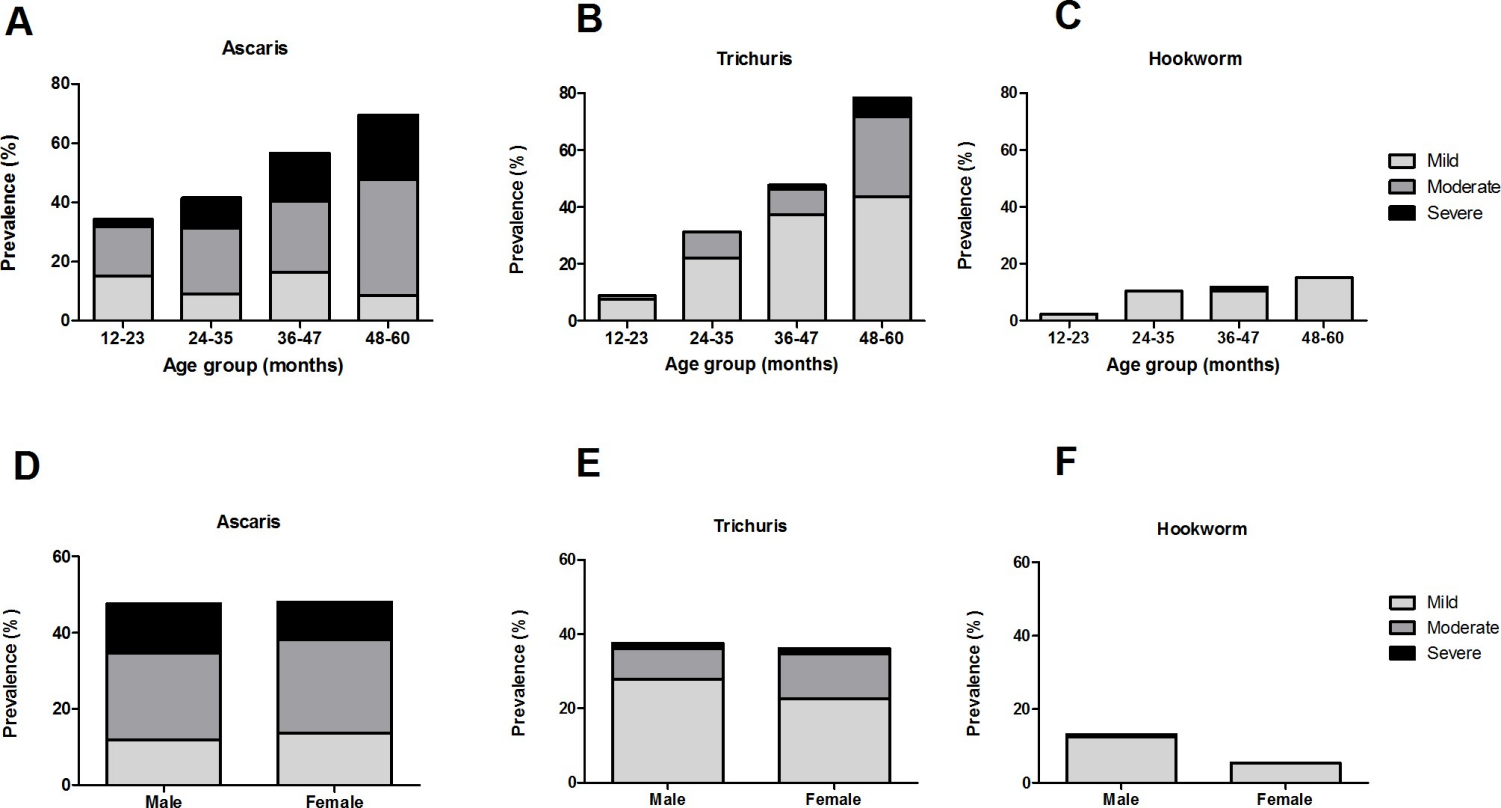
Prevalence and intensity of (A) *A*. *lumbricoides*, (B) *T*. *trichiura*, and (C) hookworm infections based on age groups, and (D) *A*. *lumbricoides*, (E) *T*. *trichiura*, and (F) hookworm infections based on sex.

STH infections were found in 58.8% out of 272 PSC, with majority of the infections were caused by *A*. *lumbricoides* (47.4%), followed by *T*. *trichiura* (36.8%) and hookworm (9.2%; **Table 1).** Co-infections were observed in 78 PSC (28.7%): 20.6% were co-infected with *A*. *lumbricoides* and *T*. *trichiura* while 2.2% were co-infected with *A*. *lumbricoides* and hookworm. The remaining 16 PSC (5.9%) were co-infected with all three STH species. *T*. *trichiura* and hookworm infections were mostly mild (68.0% and 96.0%, respectively), while a majority of *A*. *lumbricoides* infections were moderate (49.6%). The prevalence of STH infection increased with age (*A*. *lumbricoides* and *T*. *trichiura*: p<0.001; hookworm: p = 0.065; **S1 Table**). The trend was seen especially in moderate and severe intensity of *A*. *lumbricoides* infection **(Fig 1A)**, all intensity levels of *T*. *trichiura* infection **(Fig 1B)**, and mild intensity of hookworm infection **(Fig 1C).** Severe hookworm infection was only found in a 46-month-old PSC. In relation to sex, hookworm infections were more prevalent in boys than girls (13.2% vs. 5.1%, p = 0.021), while *A*. *lumbricoides* and *T*. *trichiura* infections were similar in both sexes (*A*. *lumbricoides*, boys vs. girls: 46.3% vs. 48.5%, p = 0.716, respectively; *T*. *trichiura*, both sexes 36.8%, p = 1.000; **S1 Table**). Moderate *A*. *lumbricoides* and *T*. *trichiura* infections were more prevalent in girls than boys (24.8% vs 22.8% and 12.0% vs 8.1%, respectively); however, severe infections especially of *A*. *lumbricoides* were more common in boys than girls (13.2% vs 9.8%) **(Fig 1D and 1E)**. When analyzed with Chi-squared tests, the distribution of infection intensity (**S1 Table**) was not significantly different between sexes and among age groups (p>0.05), except for the severity trend which were increasing with age (Linear-by-linear association; *A*.*lumbricoides*: p = 0.018, *T*. *trichiura*: p = 0.079, respectively).

### Nutritional status and anemia status

Anthropometric findings showed that 33.1% out of 393 PSC were underweight or severely underweight, 40.2% were stunted or severely stunted and 17.1% were wasted or severely wasted **(Table 1)**. Underweight/severely underweight and stunting/severely stunting were more prevalent in boys than girls (37.9% vs. 27.8%, p = 0.034 and 45.6% vs. 34.2%, p = 0.021, respectively, **Table 2**). With regards to the distribution of underweight and severely underweight, as well as those in the other two anthropometric parameters, no significant associations with sex and age group were found (p>0.05).

**Table 2:** Distribution of preschool-age children according to z-scores of anthropometric measurements.

Z-score	Boys, n(%)			Girls, n(%)		
	Weight-for-age	Height-for-age	Weight-for-height	Weight-for-age	Height-for-age	Weight-for-height
< -3	17 (8.3)	34 (16.5)	5 (2.4)	17 (9.1)	21 (11.2)	6 (3.2)
-3 to -2	61 (29.6)	60 (29.1)	26 (12.6)	35 (18.7)	43 (23.0)	30 (16.1)
≥-2	128 (62.1)	112 (54.4)	175 (85.0)	135 (72.2)	123 (65.8)	150 (80.6)
Total	206 (100)	206 (100)	206 (100)	187 (100)	187 (100)	186 (99.5)

About 60.3% PSC suffered from anemia **(Table 1)**. The prevalence of anemia decreased with increasing age (p<0.001), while its prevalence was relatively similar in both sexes (61.1% in boys and 59.5% in girls, p = 0.806).

### Association between STH and nutritional or anemia status

Among the STH-infected PSC, 29.4% were underweight, 35.6% were stunted, 15.6% were wasted, and 50.5% were anemic. In contrast, the prevalence of underweight, stunting, wasting, and anemia in healthy PSC were 33.0%, 46.4%, 17.9%, and 75.4%, respectively.

Single STH infection was associated with a lower risk of stunting compared to non-infected group (OR 0.506 [95% CI: 0.278–0.921], p = 0.026; **Table 3**), and this effect was more prominent in moderate STH infection (vs no infection: OR 0.508 [95% CI: 0.272, 0.948], p = 0.033; **S2 Table**). No significant effect on stunting was found for multiple STH infection as well as for mild or severe infection. Older age and girls had less risk of stunting (age: 36–47 [OR 0.543, 95% CI: 0.309–0.954, p = 0.034] and 48–60 [OR 0.474, 95% CI: 0.255–0.880, p = 0.018]; sex: girls [OR 0.620, 95% CI: 0.412–0.932, p = 0.022]). Other variables were not significantly associated with stunting (p>0.20). After adjusting for potential confounders, only child age remained a significant determinant for stunting (36–47 months, adjusted OR [aOR] 0.428 [95% CI: 0.210–0.875], p = 0.020; 48–60 months, aOR 0.315 [95% CI: 0.132–0.749], p = 0.009).

**Table 3:** Factors associated with height-for-age Z-score (HAZ) among preschool-age children (PSC) in this study (n = 393).

Variables		N (%)	Univariate			Multivariate		
			OR	95% CI	P-value	aOR	95% CI	P-value
Age (months)								
	12–23 (n = 120)	59 (49.2)	ref			ref		
	24–35 (n = 113)	46 (40.7)	0.710	[0.423, 1.192]	0.195	0.542	[0.283, 1.036]	0.064
	36–47 (n = 90)	31 (34.4)	0.543	[0.309, 0.956]	**0.034**	0.428	[0.210, 0.875]	**0.020**
	48–60 (n = 70)	22 (31.4)	0.474	[0.255, 0.880]	**0.018**	0.315	[0.132, 0.749]	**0.009**
Sex								
	Boys (n = 206)	94 (45.6)	ref			ref		
	Girls (n = 187)	64 (34.2)	0.620	[0.412, 0.932]	**0.022**	0.869	[0.524, 1.441]	0.587
Maternal education								
	None (n = 41)	17 (41.5)	1.259	[0.451, 3.514]	0.660			
	Primary (n = 165)	77 (46.7)	1.556	[0.650, 3.721]	0.321			
	Secondary (n = 161)	55 (34.2)	0.922	[0.383, 2.222]	0.857			
	Higher education (n = 25)	9 (36.0)	ref					
Co-infection								
	No infection (n = 112)	52 (46.4)	ref			ref		
	Single (n = 82)	25 (30.5)	0.506	[0.278, 0.921]	**0.026**	0.595	[0.321, 1.105]	0.100
	Multiple (n = 78)	32 (41.0)	0.803	[0.447, 1.440]	0.461	1.231	[0.633, 2.396]	0.540
Maternal anemia status								
	Anemia (n = 111)	42 (37.8)	0.855	[0.500, 1.461]	0.566			
	Normal (n = 113)	47 (41.6)	ref					
Breastfeeding status								
	Never (n = 10)	4 (40.0)	1.018	[0.280, 3.701]	0.979			
	Exclusive + Timely complementary feeding (n = 240)	95 (39.6)	ref					
	Exclusive + Late complementary feeding (n = 30)	15 (50.0)	1.526	[0.713, 3.267]	0.276			
	Non-exclusive (n = 94)	34 (36.2)	0.865	[0.528, 1.417]	0.565			

OR, odds ratio; aOR, adjusted odds ratio.

In contrast to stunting, only sex was associated with underweight in univariate analysis (girls vs boys, OR 0.632 [95% CI: 0.413–0.968], p = 0.035). In the multivariable analyses, no variables were independently associated with underweight or wasting **(Table 4 and Table 5,** respectively**).**

**Table 4:** Factors associated with weight-for-age Z-score (WAZ) among preschool-age children (PSC) in this study (n = 393).

Variables		N (%)	Univariate			Multivariate		
			OR	95% CI	P-value	aOR	95% CI	P-value
Age (months)								
	12–23 (n = 120)	35 (29.2)	ref			ref		
	24–35 (n = 113)	37 (32.7)	1.155	[0.661, 2.015]	0.613	1.177	[0.659, 2.103]	0.582
	36–47 (n = 90)	33 (36.7)	1.373	[0.766, 2.459]	0.287	1.266	[0.687, 2.334]	0.450
	48–60 (n = 70)	25 (35.7)	1.317	[0.703, 2.470]	0.390	1.391	[0.723, 2.676]	0.323
Sex								
	Boys (n = 206)	78 (37.9)	ref			ref		
	Girls (n = 187)	52 (27.8)	0.632	[0.413, 0.968]	**0.035**	0.734	[0.471, 1.144]	0.173
Maternal education								
	None (n = 41)	15 (36.6)	1.026	[0.364, 2.887]	0.962			
	Primary (n = 165)	58 (35.2)	0.964	[0.401, 2.316]	0.934			
	Secondary (n = 161)	48 (29.8)	0.755	[0.312, 1.827]	0.533			
	Higher education (n = 25)	9 (36.0)	ref					
Co-infection								
	No infection (n = 112)	37 (33.0)	ref					
	Single (n = 82)	25 (30.5)	0.889	[0.481, 1.642]	0.707			
	Multiple (n = 78)	22 (28.2)	0.796	[0.424, 1.497]	0.479			
Maternal anemia status								
	Anemia (n = 111)	34 (30.6)	0.871	[0.497, 1.528]	0.631			
	Normal (n = 113)	38 (33.6)	ref					
Breastfeeding status								
	Never (n = 10)	6 (60.0)	2.890	[0.793, 10.531]	0.108	2.519	[0.679, 9.344]	0.167
	Exclusive + Timely complementary feeding (n = 240)	82 (34.2)	ref			ref		
	Exclusive + Late complementary feeding (n = 30)	10 (33.3)	0.963	[0.431, 2.154]	0.928	0.987	[0.437, 2.231]	0.975
	Non-exclusive (n = 94)	26 (27.7)	0.737	[0.436, 1.245]	0.254	0.734	[0.431, 1.250]	0.256

OR, odds ratio; aOR, adjusted odds ratio.

**Table 5:** Factors associated with weight-for-height Z-score (WHZ) among preschool-age children (PSC) in this study (n = 392).

Variables		N (%)	Univariate			Multivariate		
			OR	95% CI	P-value	aOR	95% CI	P-value
Age (months)								
	12–23 (n = 120)	23 (19.2)	ref			ref		
	24–35 (n = 113)	19 (16.8)	0.835	[0.427, 1.633]	0.598	0.857	[0.437, 1.682]	0.654
	36–47 (n = 89)	12 (13.5)	0.644	[0.301, 1.376]	0.256	0.673	[0.313, 1.448]	0.311
	48–60 (n = 70)	13 (18.6)	0.942	[0.443, 2.005]	0.877	0.942	[0.442, 2.006]	0.876
Sex								
	Boys (n = 206)	31 (15.0)	ref			ref		
	Girls (n = 186)	36 (19.4)	1.355	[0.800, 2.296]	0.259	1.305	[0.766, 2.225]	0.327
Maternal education								
	No education (n = 41)	5 (12.2)	0.440	[0.119, 1.631]	0.219			
	Primary (n = 165)	31 (18.8)	0.733	[0.270, 1.986]	0.541			
	Secondary (n = 160)	25 (15.6)	0.586	[0.213, 1.614]	0.301			
	Higher education (n = 25)	6 (24.0)	ref					
Co-infections								
	No infection (n = 112)	20 (17.9)	ref					
	Single (n = 82)	16 (19.5)	1.115	[0.538, 2.313]	0.770			
	Multiple (n = 78)	9 (11.5)	0.600	[0.257, 1.399]	0.237			
Maternal anemia status								
	Anemia (n = 111)	16 (14.4)	0.889	[0.428, 1.846]	0.752			
	Normal (n = 113)	18 (15.9)	ref					
Breastfeeding status								
	Never (n = 10)	2 (20.0)	1.140	[0.234, 5.556]	0.872			
	Exclusive + Timely complementary feeding (n = 239)	43 (18.0)	ref					
	Exclusive + Late complementary feeding (n = 30)	5 (16.7)	0.912	[0.330, 2.516]	0.858			
	Non-exclusive (n = 94)	15 (16.0)	0.865	[0.455, 1.647]	0.660			

OR, odds ratio; aOR, adjusted odds ratio.

We discovered that the presence of either single or multiple STH infection and older PSC (24 months and above) were associated with a lower risk of anemia. With regards to STH species, PSC infected with *A*. *lumbricoides* had a lower risk of anemia (OR 0.478, 95% CI [95% CI: 0.250–0.915], p = 0.026; **Table 6**); whereas other species did not exhibit such trend (**S3 Table**). In addition, the inverse association was also observed especially in mild and moderate STH infection (vs no infection: OR 0.275 [95% CI: 0.113–0.665], p = 0.004, and OR 0.373 [95% CI: 0.163–0.851], p = 0.019, respectively; **S2 Table**). In contrast, lower maternal education level and the presence of maternal anemia resulted in a higher risk of anemia in PSC. After adjusting for covariates, only age (24–35 months, aOR 0.209 [95% CI: 0.070–0.623], p = 0.005; 36–47 vs 12–23 months, aOR 0.216 [95% CI: 0.072–0.650], p = 0.006; 48–60 vs 12–23 months, aOR 0.175 [95% CI: 0.049–0.624], p = 0.007) and single STH infection remained significant (aOR 0.320 [95% CI: 0.126–0.809], p = 0.016; **Table 6**). Mild STH infection was also consistently associated with a lower anemia risk (OR 0.318 [95% CI: 0.114–0.887], p = 0.029; **S2 Table**).

**Table 6:** Factors associated with anemia among preschool-age children (PSC) in this study (n = 224).

Variables		N (%)	Univariate			Multivariate		
			OR	95% CI	P-value	aOR	95% CI	P-value
Age (months)								
	12–23 (n = 69)	57 (82.6)	ref			ref		
	24–35 (n = 65)	35 (53.8)	0.250	[0.113, 0.552]	**0.001**	0.209	[0.070, 0.623]	**0.005**
	36–47 (n = 53)	25 (47.2)	0.191	[0.084, 0.436]	**<0.001**	0.216	[0.072, 0.650]	**0.006**
	48–60 (n = 37)	18 (48.6)	0.203	[0.083, 0.498]	**<0.001**	0.175	[0.049, 0.624]	**0.007**
Sex								
	Boys (n = 113)	69 (61.1)	ref			ref		
	Girls (n = 111)	66 (59.5)	0.935	[0.548, 1.597]	0.806	0.700	[0.326, 1.504]	0.361
Maternal education								
	None (n = 25)	17 (68.0)	3.453	[1.022, 11.665]	**0.046**	0.643	[0.127, 3.248]	0.593
	Primary (n = 93)	63 (67.7)	3.412	[1.278, 9.112]	**0.014**	1.412	[0.371, 5.371]	0.613
	Secondary (n = 85)	47 (55.3)	2.010	[0.755, 5.350]	0.162	0.813	[0.220, 3.006]	0.756
	Higher education (n = 21)	8 (38.1)	ref			ref		
Co-infections								
	No infection (n = 61)	46 (75.4)	ref			ref		
	Single (n = 51)	24 (47.1)	0.290	[0.130, 0.646]	**0.002**	0.320	[0.126, 0.809]	**0.016**
	Multiple (n = 46)	25 (54.3)	0.388	[0.171, 0.883]	**0.024**	0.711	[0.262, 1.928]	0.503
Maternal anemia status								
	Anemia (n = 111)	76 (68.5)	1.987	[1.153, 3.426]	**0.013**	1.625	[0.752, 3.511]	0.217
	Normal (n = 113)	59 (52.2)	ref			ref		
Breastfeeding status								
	Never (n = 4)	1 (25.0)	0.201	[0.020, 1.987]	0.170	1.568	[0.065, 38.044]	0.782
	Exclusive + Timely complementary feeding (n = 125)	78 (62.4)	ref			ref		
	Exclusive + Late complementary feeding (n = 17)	14 (82.4)	2.812	[0.768, 10.302]	0.119	2.484	[0.557, 11.073]	0.233
	Non-exclusive (n = 68)	40 (58.8)	0.861	[0.471, 1.574]	0.626	0.877	[0.390, 1.969]	0.750

OR, odds ratio; aOR, adjusted odds ratio.

## Discussion

This study revealed a high prevalence of STH infections in preschool-age children (PSC) in Nangapanda, Ende, East Nusa Tenggara, with *A*. *lumbricoides* and *T*. *trichiura* as the predominant species. Our findings were in accordance to a previous study conducted in Southwest Sumba, a neighboring area of Nangapanda subdistrict, which also reported a high prevalence of these two species [21]. A national survey in 2008 further corroborated our findings by that up to 61% of Indonesia’s population were infected with STH, with ascariasis and trichuriasis being the most prevalent infections (more than 90 million and 60 million cases, respectively) [22]. The high prevalence of *A*. *lumbricoides* and *T*. *trichiura* infections observed in this study indicated that oral-fecal transmission was more common than transmission by skin penetration of hookworms, especially in children. *A*. *lumbricoides* absorbs nutrients from the host’s gut lumen, while *T*. *trichiura* lives from sucking the host’s blood. With these parasitic mechanisms, moderate-to-severe STH infections may lead to malnutrition and anemia which may have devastating impacts on child growth and cognitive performance [23].

In the present study, STH infections were more prevalent and severe in older age groups. The study by Ojja et al in Uganda also showed similar findings [24]. However, in the study, no significant correlation between age and intensity of STH infections were observed for *A*. *lumbricoides* and *T*. *trichiura* infections [24], contrary to our findings where more severe *A*. *lumbricoides* and *T*. *trichiura* infections occurred in older PSC. With regards to sex, we discovered that boys were at a higher risk of contracting hookworm infection than girls, which was also supported by the study in Uganda [24]. The fact that older PSC and boys were at higher risks of contracting hookworm infection may be due to behavioral factors exposing them to contaminated soils [24,25].

This study revealed a high proportion of malnourished PSC. Based on the classification of malnutrition severity assessment according to WHO [26], the prevalence of underweight (33.1%), stunting (40.2%), and wasting (17.1%) in this study was found to be very high (cut-off, underweight ≥30%, stunting ≥40%, wasting ≥15%). The prevalence of malnutrition in this study area was also similar to that of Indonesia National Health Survey’s finding in East Nusa Tenggara (underweight 29.5%, stunting 42.7%, wasting 12.8%). At the same time, the difficult access to health centers and/or midwives (81.9%), lack of access to water source (13.8%, 2^nd^ highest in Indonesia), as well as poor sanitation (87.3% poor household waste, 45.1% hazardous fecal waste managements) may contribute to the child’s nutritional problem [9].

We discovered that PSC at younger age was more vulnerable to stunting, even after adjusting for confounders. This finding is supported by the fact that growth delay occurs within the first two years of life and stabilizes after three years [27]. In univariate analysis, we also discovered that boys had a higher risk of stunting than girls, which is in line with the findings by Ojja et al [24]. This effect may be explained by the disparities in living conditions, sex-based biology, and breastfeeding patterns of boys due to gendered cultural perception [28]. However, since sex became an insignificant factor in the multivariate analysis, this showed that sex was not considered as a determinant for stunting in our studied children. A possible explanation is that both sexes in this study population were exposed to similar environment and upbringing, which is concurrent with previous literature [20,29]. Likewise, no significant correlation with stunting was seen in single or multiple STH infection after adjustment for other factors. This finding is supported by previous studies in which the presence of STH were not associated with stunting [30–32].

The prevalence of anemia in this study was 60.3%, which was similar to previous studies conducted in neighboring areas (Northwest Sumba, 57.1%; Southwest Sumba, 71.2%) [33,34] and remarkably higher than that reported in the Indonesian National Health Survey in 2013 (28.1%) [12], implying that anemia in this area requires immediate attention.

In this study, we demonstrated that maternal anemia and lower maternal education level were significantly associated with a higher risk of anemia, while the opposite was seen for older PSC and STH infection. After adjusting for confounders, age and single STH infection remained significant, which was concordant with the findings of previous studies [35–37]. Interistingly, we found that single STH infection was independently associated with lower risk of anemia. Although several studies have shown that STH infection was an independent predictor of anemia [38,39], the observed trend remained equivocal as previous studies in Indonesia revealed that STH infections were not associated with anemia [40,41]. Our findings were similar to those in the study by Knopp et al, showing that STH infection, especially *A*. *lumbricoides* as the predominant species, was associated with lower risk of anemia [42].

Further analysis showed that the inverse association between STH infection and anemia was accentuated in mild STH infection, whereas the same association in moderate or severe STH infection was non-significant. It is known that higher STH severity causes greater morbidity as the STH-induced blood loss outweighs the iron reserves and the dietary iron intake [43,44]. In our study, most of the STH infections were mild-to-moderate, suggesting that the blood loss caused by STH infection may be subtle. Moreover, the prevalence of hookworm infection, which is a well-known risk factor of anemia compared to other STH species [44], were relatively low in this study. Most hookworm infections were also caused by *Necator americanus* (75%) rather than *Ancylostoma duodenale* (6.4%) [10], which causes more blood loss [44]. Another possible explanation is that although the non-infected children at the time of study had no or undetected STH infection, they might have harbored STH infection in the past that could cause detrimental effects on their nutritional status even after the infection resolved. This premise could be proven by following the children up in a longitudinal fashion through serial stool examinations and data collection on the history of anthelminthic administration. These factors may potentially explain the trend observed in this study.

The decreasing trend of anemia in older PSC may be elaborated by the high nutritional demand due to accelerated growth rate during early years of life and the rapidly expanding blood volume [37], which could be compensated later in older age. In addition, PSC in developing countries, including Indonesia, are usually given rice-based complementary foods which contain low micronutrient contents, notably iron, thus predisposing these children to iron-deficiency anemia [37]. These premises may also explain the cause of high rate of iron-deficiency anemia in younger PSC in Indonesia, which averaged about 61.3%, 64.8%, and 48.1% of PSC aged 0–6, 6–12, and 12–60 months, respectively [45].

### Study strengths and limitations

The strength of this study relies on the relatively large number of PSC in the Nangapanda subdistrict; thus, allowing us to explore the effects of different STH species as well as multiple infections on nutritional and anemia status. Although we were unable to establish a firm evidence linking STH infection with poor nutritional status, our study revealed a very high prevalence of STH infection, malnutrition, and anemia among these age groups in this area, hence warranting immediate actions to control STH infections and to improve the nutritional status of these PSC. This is saliently important, considering that the first 1000 days of life is crucial to the child’s growth and development [46].

With the stunting prevalence of 40% and the anemia prevalence of 60%, the sample size in this study was still adequate; however, it should be noted that only 272 (69.2%) PSC had data on STH infection, thereby suggesting that this study was underpowered to detect significant associations between STH infection and stunting or anemia. On the other hand, the inverse association that was significantly found between single and mild STH infections with anemia, even after adjusting for potential confounders, may reflect the real situation in this PSC population which needs further investigation. This study was limited due to its cross-sectional design, therefore implying the inability to explain causalities between variables. Furthermore, there are possibilities that other factors not reported in the present study might confound the observed relationships on the nutritional and anemia status, such as bacterial or viral infection related to poor sanitation or hygiene behavior, and the quality and quantity of nutrient intake. Although our study did not investigate other parasitic infections that are known to cause malnutrition and anemia such as malaria or other helminths which were endemic in Indonesia,[22], a previous study has shown that the prevalence of malaria among schoolchildren in the same study area in Nangapanda was only 5.8% by real-time PCR [47], and that no other species of helminth was detected by microscopic examination[5], indicating that the potential effects of these confounders may be negligible. In addition, the possible confounding effects of intestinal protozoan infections such as *Giardia lamblia* or *Entamoeba histolytica* were less likely as the stool samples were collected from non-diarrheic PSC. Nonetheless, our findings should still be interpreted cautiously as the fact that no factors were associated with underweight and wasting suggested that there were unexplored risk factors in the study population.

## Conclusion

In summary, the study revealed that preschool-age children in this area was severely burdened by STH infection (especially *A*. *lumbricoides* and *T*. *trichiura*), malnutrition, and anemia. However, the high prevalence of STH infection during early childhood in the present study area was not associated with poorer nutritional status. In fact, single and mild STH infection was independently associated with a lower risk of anemia, while older age was associated with lower risks of stunting and anemia. Future investigations on the potential interplay between STH and other concurrent infections, nutrient intake, as well as the environmental and socio-cultural factors contributing to nutritional deficiencies of the children in this region are urgently needed.

## Supporting information

S1 Table Association between age and sex with frequency and intensity of soil-transmitted helminth infection. (DOCX)

S2 Table Association between intensity of soil-transmitted helminth infection and anthropometric or anemia status. (DOCX)

S3 Table Association between species of soil-transmitted helminth infection and anthropometric or anemia status. (DOCX)
