# Supplementary material for: Soil-transmitted helminth infection, anemia, and malnutrition among preschool-age children in Nangapanda subdistrict, Indonesia
Source: PLoS Negl Trop Dis. 2021 Jun 17;15(6):e0009506. doi: 10.1371/journal.pntd.0009506 (PMC8253427; doi:10.1371/journal.pntd.0009506)
Supplement: S1 Table — (DOCX) [file pntd.0009506.s001.docx]

S1 Table. Association between age and sex with prevalence and intensity of soil-transmitted helminth infection

| **Variables** | **Total (n=272)** | **Prevalence** | | | **Intensity** | | | |
| --- | --- | --- | --- | --- | --- | --- | --- | --- |
|  |  | **Infected** | **Uninfected** | **P-value** | **Mild** | **Moderate** | **Severe** | **P-value** |
| ***A. lumbricoides*** |  |  |  |  |  |  |  |  |
| Age (months) |  |  |  |  |  |  |  |  |
| 12 – 23 | 82 (30.1) | 27 (32.9) | 55 (67.1) | <0.001 | 11 (40.7) | 13 (48.1) | 3 (11.1) | 0.200 |
| 24 – 35 | 77 (28.3) | 32 (41.6) | 45 (58.4) |  | 7 (21.9) | 17 (53.1) | 8 (25.0) |  |
| 36 – 47 | 67 (24.6) | 38 (56.7) | 29 (43.3) |  | 11 (28.9) | 16 (42.1) | 11 (28.9) |  |
| 48 – 60 | 46 (16.9) | 32 (69.6) | 14 (30.4) |  | 4 (12.5) | 18 (56.3) | 10 (31.3) |  |
| Sex |  |  |  |  |  |  |  |  |
| Boys | 136 (50.0) | 63 (46.3) | 73 (53.7) | 0.716 | 15 (23.8) | 31 (49.2) | 17 (27.0) | 0.823 |
| Girls | 136 (50.0) | 66 (48.5) | 70 (51.5) |  | 18 (27.3) | 33 (50.0) | 15 (22.7) |  |
| ***T. trichiura*** |  |  |  |  |  |  |  |  |
| Age (months) |  |  |  |  |  |  |  |  |
| 12 – 23 | 82 (30.1) | 8 (9.8) | 74 (90.2) | <0.001 | 6 (75.0) | 2 (25.0) | 0 (0) | 0.364^a^ |
| 24 – 35 | 77 (28.3) | 24 (31.2) | 53 (68.8) |  | 17 (70.8) | 7 (29.2) | 0 (0) |  |
| 36 – 47 | 67 (24.6) | 32 (47.8) | 35 (52.2) |  | 25 (78.1) | 6 (18.8) | 1 (3.1) |  |
| 48 – 60 | 46 (16.9) | 36 (78.3) | 10 (21.7) |  | 20 (55.6) | 13 (36.1) | 3 (8.3) |  |
| Sex |  |  |  |  |  |  |  |  |
| Boys | 136 (50.0) | 50 (36.8) | 86 (63.2) | 1.000 | 37 (74.0) | 11 (22.0) | 2 (4.0) | 0.397^a^ |
| Girls | 136 (50.0) | 50 (36.8) | 86 (63.2) |  | 31 (62.0) | 17 (34.0) | 2 (4.0) |  |
| **Hookworm** |  |  |  |  |  |  |  |  |
| Age (months) |  |  |  |  |  |  |  |  |
| 12 – 23 | 82 (30.1) | 2 (2.4) | 80 (97.6) | 0.065 | 2 (100) | 0 (0) | 0 (0) | 1.000^a^ |
| 24 – 35 | 77 (28.3) | 8 (10.4) | 69 (89.6) |  | 8 (100) | 0 (0) | 0 (0) |  |
| 36 – 47 | 67 (24.6) | 8 (11.9) | 59 (88.1) |  | 7 (87.5) | 0 (0) | 1 (12.5) |  |
| 48 – 60 | 46 (16.9) | 7 (15.2) | 39 (84.8) |  | 7 (100) | 0 (0) | 0 (0) |  |
| Sex |  |  |  |  |  |  |  |  |
| Boys | 136 (50.0) | 18 (13.2) | 118 (86.8) | 0.021 | 17 (94.4) | 0 (0) | 1 (5.6) | 1.000^b^ |
| Girls | 136 (50.0) | 7 (5.1) | 129 (94.9) |  | 7 (100) | 0 (0) | 0 (0) |  |

^a^The P-value was calculated using Monte-Carlo exact method with a sample size of 393. ^b^The P-value was calculated using Fisher’s exact test
