# Supplementary material for: Soil-transmitted helminth infection, anemia, and malnutrition among preschool-age children in Nangapanda subdistrict, Indonesia
Source: PLoS Negl Trop Dis. 2021 Jun 17;15(6):e0009506. doi: 10.1371/journal.pntd.0009506 (PMC8253427; doi:10.1371/journal.pntd.0009506)
Supplement: S2 Table — (DOCX) [file pntd.0009506.s002.docx]

**S2 Table.** Association between intensity of soil-transmitted helminth infection and anthropometric or anemia status

| Variables | | N (%) | Univariate | | | Age- and sex-adjusted | | | Fully adjusted | | | |
| --- | --- | --- | --- | --- | --- | --- | --- | --- | --- | --- | --- | --- |
|  |  |  | OR | 95% CI | P-value | OR | 95% CI | P-value | | OR | 95% CI | P-value |
| Underweight | |  |  |  |  |  |  |  | |  |  |  |
|  | No infection (n=112) | 37 (33.0) | ref |  |  | ref |  |  | | ref |  |  |
|  | Mild (n=54) | 16 (29.6) | 0.853 | [0.422, 1.726] | 0.659 | 0.807 | [0.392, 1.662] | 0.560 | | 0.916^a^ | [0.435, 1.926] | 0.817 |
|  | Moderate (n=72) | 20 (27.8) | 0.780 | [0.407, 1.492] | 0.452 | 0.688 | [0.341, 1.389] | 0.297 | | 0.796^a^ | [0.389, 1.631] | 0.533 |
|  | Severe (n=34) | 11 (32.4) | 0.969 | [0.427, 2.200] | 0.941 | 0.825 | [0.344, 1.978] | 0.666 | | 0.942^a^ | [0.378, 2.343] | 0.897 |
| Stunting | |  |  |  |  |  |  |  | |  |  |  |
|  | No infection (n=112) | 52 (46.4) | ref |  |  | ref |  |  | | ref |  |  |
|  | Mild (n=54) | 23 (42.6) | 0.856 | [0.445, 1.648] | 0.642 | 0.993 | [0.506, 1.952] | 0.984 | | 0.993^b^ | [0.506, 1.952] | 0.984 |
|  | Moderate (n=72) | 22 (30.6) | 0.508 | [0.272, 0.948] | 0.033 | 0.646 | [0.331, 1.261] | 0.200 | | 0.646^b^ | [0.331, 1.261] | 0.200 |
|  | Severe (n=34) | 12 (35.3) | 0.629 | [0.284, 1.394] | 0.254 | 0.858 | [0.368, 2.001] | 0.722 | | 0.858^b^ | [0.368, 2.001] | 0.722 |
| Wasting | |  |  |  |  |  |  |  | |  |  |  |
|  | No infection (n=112) | 20 (17.9) | ref |  |  | ref |  |  | | ref |  |  |
|  | Mild (n=54) | 8 (14.8) | 0.800 | [0.328, 1.954] | 0.624 | 0.771 | [0.309, 1.924] | 0.577 | | 0.771^b^ | [0.309, 1.924] | 0.577 |
|  | Moderate (n=72) | 12 (16.7) | 0.920 | [0.419, 2.019] | 0.835 | 0.810 | [0.343, 1.911] | 0.630 | | 0.810^b^ | [0.343, 1.911] | 0.630 |
|  | Severe (n=34) | 5 (14.7) | 0.793 | [0.273, 2.301] | 0.670 | 0.718 | [0.232, 2.224] | 0.566 | | 0.718^b^ | [0.232, 2.224] | 0.566 |
| Anemia | |  |  |  |  |  |  |  | |  |  |  |
|  | No infection (n=61) | 46 (75.4) | ref |  |  | ref |  |  | | ref |  |  |
|  | Mild (n=35) | 16 (45.7) | 0.275 | [0.113, 0.665] | 0.004 | 0.340 | [0.130, 0.890] | 0.028 | | 0.318^c^ | [0.114, 0.887] | 0.029 |
|  | Moderate (n=45) | 24 (53.3) | 0.373 | [0.163, 0.851] | 0.019 | 0.561 | [0.223, 1.410] | 0.219 | | 0.619^c^ | [0.228, 1.681] | 0.347 |
|  | Severe (n=17) | 9 (52.9) | 0.367 | [0.120, 1.120] | 0.078 | 0.608 | [0.182, 2.037] | 0.420 | | 0.442^c^ | [0.122, 1.602] | 0.214 |

^a^Adjusted by age, sex, and breastfeeding status. ^b^Adjusted by age and sex. ^c^Adjusted by age, sex, maternal education level, maternal anemia status, and breastfeeding status. CI, confidence interval; OR, odds ratio.
