# Supplementary material for: Soil-transmitted helminth infection, anemia, and malnutrition among preschool-age children in Nangapanda subdistrict, Indonesia
Source: PLoS Negl Trop Dis. 2021 Jun 17;15(6):e0009506. doi: 10.1371/journal.pntd.0009506 (PMC8253427; doi:10.1371/journal.pntd.0009506)
Supplement: S3 Table — (DOCX) [file pntd.0009506.s003.docx]

**S3 Table.** Association between species of soil-transmitted helminth and anthropometric or anemia status

| Variables | | N (%) | | Univariate | | | | Age- and sex-adjusted | | | | Fully adjusted | | |
| --- | --- | --- | --- | --- | --- | --- | --- | --- | --- | --- | --- | --- | --- | --- |
|  |  | Infected | Uninfected | OR | 95% CI | P-value | OR | | 95% CI | P-value | OR | | 95% CI | P-value |
| Underweight | |  |  |  |  |  |  | |  |  |  | |  |  |
|  | *A. lumbricoides* | 39 (30.2) | 45 (31.5) | 0.944 | [0.564, 1.580] | 0.826 | 0.899 | | [0.524, 1.540] | 0.697 | 0.936^a^ | | [0.535, 1.638] | 0.817 |
|  | *T. trichiura* | 29 (29.0) | 55 (32.0) | 0.869 | [0.507, 1.488] | 0.609 | 0.729 | | [0.394, 1.350] | 0.315 | 0.797^a^ | | [0.422, 1.506] | 0.485 |
|  | Hookworm | 7 (28.0) | 77 (31.2) | 0.859 | [0.344, 2.141] | 0.744 | 0.736 | | [0.289, 1.874] | 0.521 | 0.758^a^ | | [0.291, 1.971] | 0.569 |
| Stunting | |  |  |  |  |  |  | |  |  |  | |  |  |
|  | *A. lumbricoides* | 48 (37.2) | 61 (42.7) | 0.797 | [0.489, 1.297] | 0.360 | 0.958 | | [0.547, 1.600] | 0.870 | 0.958^b^ | | [0.547, 1.600] | 0.870 |
|  | *T. trichiura* | 38 (38.0) | 71 (41.3) | 0.872 | [0.526, 1.445] | 0.595 | 1.346 | | [0.745, 2.435] | 0.325 | 1.346^b^ | | [0.745, 2.435] | 0.325 |
|  | Hookworm | 9 (36.0) | 100 (40.5) | 0.827 | [0.352, 1.945] | 0.663 | 0.976 | | [0.402, 2.368] | 0.957 | 0.976^b^ | | [0.402, 2.368] | 0.957 |
| Wasting | |  |  |  |  |  |  | |  |  |  | |  |  |
|  | *A. lumbricoides* | 20 (15.5) | 25 (17.5) | 0.866 | [0.455, 1.647] | 0.661 | 0.829 | | [0.424, 1.620] | 0.583 | 0.829^b^ | | [0.424, 1.620] | 0.583 |
|  | *T. trichiura* | 13 (13.0) | 32 (18.6) | 0.654 | [0.325, 1.314] | 0.233 | 0.515 | | [0.229, 1.159] | 0.109 | 0.515^b^ | | [0.229, 1.159] | 0.109 |
|  | Hookworm | 4 (16.0) | 41 (16.6) | 0.957 | [0.312, 2.935] | 0.939 | 1.014 | | [0.319, 3.218] | 0.982 | 1.014^b^ | | [0.319, 3.218] | 0.982 |
| Anemia | |  |  |  |  |  |  | |  |  |  | |  |  |
|  | *A. lumbricoides* | 40 (51.3) | 55 (68.8) | 0.478 | [0.250, 0.915] | 0.026 | 0.638 | | [0.316, 1.287] | 0.209 | 0.642^c^ | | [0.305, 1.352] | 0.244 |
|  | *T. trichiura* | 33 (54.1) | 62 (63.9) | 0.665 | [0.347, 1.277] | 0.221 | 1.204 | | [0.560, 2.589] | 0.634 | 1.300^c^ | | [0.579, 2.923] | 0.525 |
|  | Hookworm | 6 (50.0) | 89 (61.0) | 0.640 | [0.197, 2.083] | 0.459 | 0.888 | | [0.256, 3.077] | 0.851 | 1.051^c^ | | [0.285, 3.875] | 0.940 |

^a^Adjusted by age, sex, and breastfeeding status. ^b^Adjusted by age and sex. ^c^Adjusted by age, sex, maternal education level, maternal anemia status, and breastfeeding status. CI, confidence interval; OR, odds ratio.
